# CAR-T Cell Performance: How to Improve Their Persistence?

**DOI:** 10.3389/fimmu.2022.878209

**Published:** 2022-04-28

**Authors:** Gina López-Cantillo, Claudia Urueña, Bernardo Armando Camacho, Cesar Ramírez-Segura

**Affiliations:** ^1^ Laboratorio de Investigación en Ingeniería Celular y Molecular, Instituto Distrital de Ciencia Biotecnología e Innovación en Salud (IDCBIS), Bogotá, Colombia; ^2^ Grupo de Inmunobiología y Biología Celular, Facultad de Ciencias, Pontificia Universidad Javeriana, Bogotá, Colombia; ^3^ Instituto Distrital de Ciencia Biotecnología e Innovación en Salud (IDCBIS), Bogotá, Colombia

**Keywords:** CAR-T cells, persistence, metabolism, differentiation status, culture optimization

## Abstract

Adoptive cell therapy with T cells reprogrammed to express chimeric antigen receptors (CAR-T cells) has been highly successful in patients with hematological neoplasms. However, its therapeutic benefits have been limited in solid tumor cases. Even those patients who respond to this immunotherapy remain at risk of relapse due to the short-term persistence or non-expansion of CAR-T cells; moreover, the hostile tumor microenvironment (TME) leads to the dysfunction of these cells after reinfusion. Some research has shown that, in adoptive T-cell therapies, the presence of less differentiated T-cell subsets within the infusion product is associated with better clinical outcomes. Naive and memory T cells persist longer and exhibit greater antitumor activity than effector T cells. Therefore, new methods are being studied to overcome the limitations of this therapy to generate CAR-T cells with these ideal phenotypes. In this paper, we review the characteristics of T-cell subsets and their implications in the clinical outcomes of adoptive therapy with CAR-T cells. In addition, we describe some strategies developed to overcome the reduced persistence of CAR T-cells and alternatives to improve this therapy by increasing the expansion ability and longevity of modified T cells. These methods include cell culture optimization, incorporating homeostatic cytokines during the expansion phase of manufacturing, modulation of CAR-T cell metabolism, manipulating signaling pathways involved in T-cell differentiation, and strategies related to CAR construct designs.

## Introduction

On August 30, 2017, the US Food and Drug Administration (FDA) approved the use of tisagenlecleucel (CTL-019; Kymriah^®^, Novartis, Basel, Switzerland), a CD19-directed chimeric antigen receptor (CAR) T cell, for adoptive cell therapy (ACT) to treat patients with relapsed/refractory B-cell acute lymphoblastic leukemia (r/r B-ALL) (1–3). The complete remission rate (CRR) was 63% [95% confidence interval (CI), 50%–75%], and all patients in complete remission (CR) attained minimal residual disease (MRD) less than 0.01% after a median follow-up of 4.8 months (2). In October 2017, the FDA granted regular approval for axicabtagene ciloleucel (Yescarta), another CAR-T cell directed against CD19, to treat adult patients with relapsed/refractory large B-cell lymphoma after two or more lines of systemic therapy (4). Efficacy was assessed in terms of CRR and duration of response in 101 adult patients with relapsed/refractory large B-cell lymphoma (median of 3 prior systemic regimens) who underwent treatment in a single-arm trial (5). In 2020, brexucabtagene autoleucel was approved for the treatment of relapsed/refractory diffuse large B-cell and mantle cell lymphomas (6, 7). Likewise, lisocabtagene maraleucel was approved in February 2021 as therapy for refractory large B-cell lymphoma (7, 8). Recently, the FDA has approved a CAR-T cell immunotherapy for multiple myeloma; the first-in-class B-cell maturation antigen (BCMA)-targeted CAR-T cell therapy received the agency’s approval on March 26, 2021, to treat adults with relapsed/refractory multiple myeloma (9, 10).

The clinical response to CAR-T cell therapy has been associated with the *in vivo* expansion and long-term persistence of functional CAR-T cells (11). Mounting evidence suggests that successful outcomes in patients treated with CAR-T cells depend on the cells’ ability to expand and persist after infusion. One of the major issues of using CAR-T cells for the treatment of solid tumors is the low persistence of the cells infused within the tumor mass (12). Long-term persistence and robust *in vivo* expansion of CAR-T cells infused during ACT are associated with sustained clinical remission and survival of recipient patients (11, 13–15). In 2019, Hay et al. reported a study on adult patients with B-ALL infused with CD19-directed CAR-T cells. Although the MRD was negative in 85% of individuals and the CRR was high, 49% of patients relapsed after CAR-T cell infusion. Before or at relapse, the CAR transgene copies were low or undetectable in peripheral blood (16).

Different factors have been associated with long-lasting remission after the adoptive transfer of CAR-T cells. One of the critical aspects that determine the efficacy of CAR-T cell therapy is the *in vivo* persistence of the cells infused. Some studies have shown that the persistence of CAR-T cells is correlated with the phenotype of the T cells infused and that prolonged detection of CAR-T cells is associated with superior responses even in patients with high-grade diseases (17). In search of determinants of therapeutic response to CD19-directed CAR-T cells in patients with chronic lymphocytic leukemia (CLL), a study evaluated the genomic and phenotypic features of the cells infused. The authors found that CAR-T cells from patients in CR exhibited upregulation of genes related to a memory cell phenotype, whereas CAR-T cells from patients with no clinical response showed upregulation of genes associated with an effector or exhausted cell phenotype (18). In 2014, Maude et al. reported a pilot clinical trial of 25 patients with r/r B-ALL treated with CD19-directed CAR-T cells. Of these patients, 90% achieved CR on day 28 and 6 months after infusion; the rate of relapse-free survival was 80%. Furthermore, they found an association between the persistence of CAR-T cells in peripheral blood and B-cell aplasia in patients who had a response (13). The recovery of CD19^+^ lymphocytes from peripheral blood within the first semester following infusion of CAR-T cells indicated the disappearance of these CAR-T cells or the loss of their function (19).

The quantification of CAR-T cells is usually performed by flow cytometry, to detect the surface expression of CAR, or by quantitative polymerase chain reaction, to detect the CAR gene, but not its expression. However, CAR detection does not imply clinical response as cells can be not functionally active (20). An indirect parameter of persistent CD19-directed CAR-T cells after adoptive transfer is B-cell aplasia (20). Several studies have shown an association between the length of cancer remission and B-cell aplasia. B-cell aplasia is an indicator of functionally active CAR-T cells that deplete CD19^+^ B cells and is associated with a sustained therapy response (11, 13).

This review focuses on the different approaches used by researchers worldwide to achieve the persistence of CAR-T cells and improve their immunophenotype for better treatment response.

## CAR-T Cell Differentiation Stage and Treatment Response

The differentiation stage of T cells affects their proliferative and survival abilities. The proliferation and the survival of adoptively transferred T cells strongly correlate with their antitumor activity (21–23). The immunophenotype of cells used to start the manufacture of CAR-T cells relates to the treatment outcomes. For instance, long-term remission is related to the enrichment of CD27^+^/CD45RO^−^/CD8^+^ T cells with memory-like features (18, 24).

The antitumor activity of adoptively transferred T cells depends on their expansion and long-term activity. Clinical results have shown that less differentiated memory T cells are required for the sustained *in vivo* persistence of adoptively transferred CAR-T cells, while naive (T_N_), central memory (T_CM_), and stem-like memory (T_SCM_) lymphocytes are related to a good response due to their ability to proliferate and live longer (25). Effector T-lymphocyte (T_E_) subsets exhibit low self-renewal ability, reduced homing to tumor niches, and lower survival than memory lymphocyte (T_M_) subpopulations (26–28). Preclinical models have been used to examine the longevity and functional features of CAR-T cells derived from memory and naive T-cell subsets. The results have demonstrated that CAR-T cells produced from the CD4^+^ and CD8^+^ T_N_ and T_CM_ subsets have greater antitumor potency and proliferation than those derived from effector memory T lymphocytes (T_EM_) (29). These data indicate that naive and memory T cells are important in CAR-T cell therapy because they display sustained proliferation and higher persistence *in vivo* (30, 31).

The diversity of the T-cell subpopulations results from the microenvironment stimulus and the cell–antigen interaction. Both CD4^+^ and CD8^+^ CAR-T cells can participate in killing malignant cells (32). Indeed, the combination of the most potent CD4^+^ and CD8^+^ CAR-expressing T-cell subsets has synergistic antitumor effects *in vivo* (29). For example, in ACT with GD2-directed CAR-T cells for the treatment of neuroblastoma, the number of CD4^+^ T cells and T_CM_ cells (CD45RO^+^/CD62L^+^) within the infused product showed high concordance with the length of persistence of CAR-T cells (17). Therefore, understanding the generation and maintenance of the different T-cell subsets is critical for proposing strategies that improve the clinical outcomes of CAR-T cell therapy (31, 33).

## T-Cell Subsets

T cells can be subdivided into several subsets identified according to the combination of molecules expressed on the cell surface (34). These phenotypic differences are related to the migratory and functional characteristics of each T-cell subpopulation (35, 36).

### Naive T Cells

**Figure 1:**
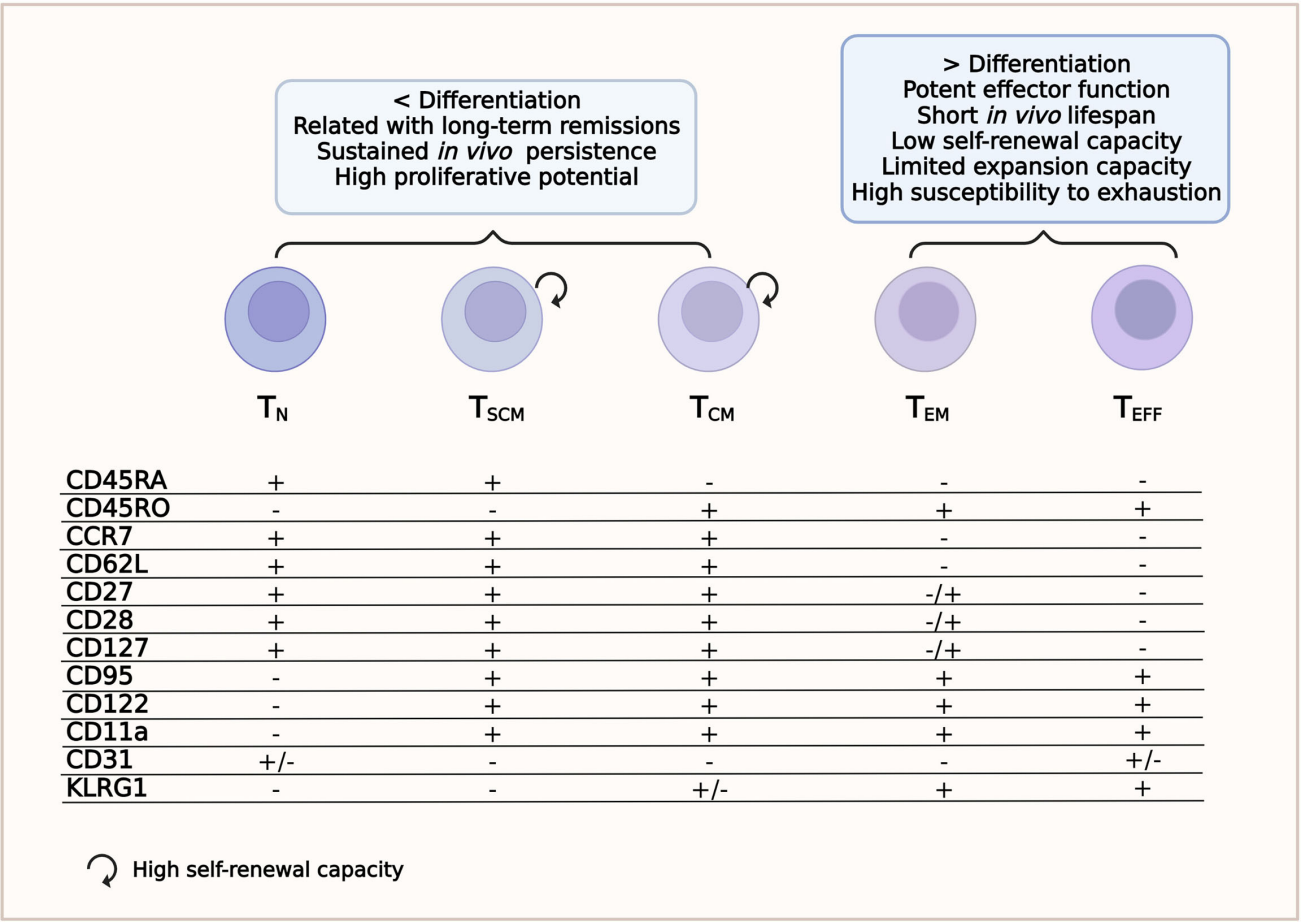
T-cell subsets. Phenotype and relevant features for a successful chimeric antigen receptor T-cell (CAR-T cell) therapy.

Immature T lymphocytes are characterized by the high expression of the transmembrane phosphatase CD45RA isoform (33, 36). Cells are considered naive until they interact with their cognate antigen. This interaction activates T_N_ lymphocytes to proliferate and differentiate into T_M_ and/or T_E_ lymphocytes (27). Other surface markers expressed by T_N_ lymphocytes are CCR7 and CD62L (l-selectin), which guide the homing of T cells to the secondary lymphoid organs; CD27 and CD28, which provide co-stimulatory signals; and CXCR4 and IL7Rα (CD127) (24, 36). The T_N_ lymphocyte subset lacks the expression of CD45RO, CD95, CD11a, CD122, CD31, and KLRG1 (**Figure 1** and **Supplementary Table S1**) **(** 28, 34, 37) and is also characterized by high proliferative ability (36).

### Stem Cell Memory T Cells

This subset is the least differentiated of the memory T-cell subpopulations. T_SCM_ cells have been recently identified as CD4^+^ or CD8^+^ T cells with a T_N_/T_M_-like phenotype. T_SCM_ cells express CD45RA, CCR7, CD95, CXCR3, CD11a, IL-2Rβ receptor (CD122), and CD127 (34, 36, 38) and have high levels of the transcriptional regulator T cell factor 1 (TCF-1), but do not express CD45RO (**Figure 1** and **Supplementary Table S1**) (37). They have a self-renewal ability similar to that of stem cells and can reconstitute all populations of T_M_ and T_E_ cells (24, 39). Compared to other T_M_ subpopulations, the T_SCM_ subset develops a faster response upon antigenic stimulation and can persist for a long time (27, 40). Because of these characteristics, the T_SCM_ subset has received significant attention in the ACT field (31). Despite their features implying several rounds of division [low expressions of T-cell receptor excision circles (TRECs) and Ki-67^high^], they maintain their naive-like phenotype (36).

### Memory T Cells with Naive Phenotypes

In 2016, Pulko et al. identified a CD8^+^ T-cell subpopulation that produced effector cytokines after stimulation through the T-cell receptor (TCR). The phenotypes of these cells were CXCR3^high^, CD49d^+^, INF-γ^+^, CD45RA^+^, CCR7^+^, CD95^low^, and CD28^int^. Although these cells shared several features with T_N_ cells, they had a restricted TCR Vβ repertoire, suggesting antigen-driven stimulation and expansion, and differed transcriptionally from T_M_ and T_E_ cells (36, 39, 41).

### Central Memory T Cells

Central memory T cells circulate through secondary lymphoid organs and are characterized by a long life span. Although T_CM_ have less cytotoxic capacity than T_E_, they can proliferate rapidly and provide an early batch of cytokines after being stimulated by the specific antigen (27, 42). They secrete tumor necrosis factor alpha (TNF-α), although more efficiently interleukin 2 (IL-2), and express CD45RO, CD62L, CD28, CD27, CCR7, CD127, CD11a, IL-18Rα, CXCR4, and CXCR3, but not CD45RA (**Figure 1** and **Supplementary Table S1**) (24, 34, 37, 43). The loss of CCR7 and CD62L accompanies the transition from T_CM_ to the T_EM_ phenotype and, therefore, the cells can no longer migrate to lymphoid tissues (27).

### Effector Memory T Cells

Effector memory T cells mainly circulate to non-lymphoid tissues and typically express CD45RO, CD122, CD95, KLRG1, LFA-1, IL-18Rα, chemokine receptors, and tissue homing receptors, but are negative for CD62L, CCR7, and CD31 (**Figure 1** and **Table Supplementary S1**) (34, 37, 43). The T_EM_ cell subset also secretes TNF-α, but has a greater capacity to release interferon gamma (IFN-γ) and are more cytotoxic than T_CM_ lymphocytes (24, 31).

### Tissue-Resident Memory T Cells

These cells are very similar to T_EM_ cells, but differ by the expression of CD103 and CD69 (33, 34). These cells do not express CD62L, CD25, CD38, and HLA-DR (34). Moreover, they remain in non-lymphoid tissues and can self-renew *in situ* and respond to secondary infections (38). Their ability to infiltrate solid tumors is well known and makes them potentially useful for developing CAR-T cells to treat these types of neoplasias (27). The surface marker expression of tissue-resident memory T cells (T_RM_) differs among tissues. On the skin, T_RM_ cells express cutaneous lymphocyte-associated antigen (CLA), CCR4, and CCR6, and about 50% of them are CCR5^+^/CXCR3^+^; in the gut, T_RM_ cells express CD69, CCR6, CCR9, and CD49d; and in the lung, they express CD49a, PSGL-1, CCR5, CXCR3, and CCR6 (34, 44).

### Terminal Effector T Cells

This cell subset comprises fully differentiated T cells ready for rapid responses and potent effector functions. However, they have a short life span and a very low self-renewal ability (27). Phenotypically, terminal effector T cells (T_EFF_) are positive for CD95, CD122, KLGR1 and several homing receptors to migrate to sites of inflammation, such as CCR5 and LFA-1; furthermore, they re-express CD45RA (34, 45). These cells do not express CD45RO, CCR7, CD62L, IL7Rα (CD127), CD27, or CD28 (**Figure 1** and **Supplementary Table S1**) (45). They also have limited expansion ability and rapidly die or become exhausted (33, 38).

### Exhausted T Cells

The persisting antigenic stimulation of antigen-specific CD8^+^ T cells throughout the responses to chronic infections or cancer leads to a gradual loss of the effector functions, with T cells becoming dysfunctional (33, 34, 38). The exhaustion features comprise the sustained expression of inhibitory receptors, altered metabolism fitness, low proliferative capacity, and a reduced secretion of effector cytokines (46). There are two main subsets of exhausted T cells (T_EX_): precursor of exhausted T cells (T_PEX_) and terminally exhausted T cells (34).

T_PEX_ have only recently been identified. This small cell subpopulation exhibits memory and exhaustion features, such as the expression of TCF-1, CD62L, ID3, and PD-1 and reduced cytokine secretion. These cells mediate the response to immune checkpoint inhibitors and can self-renew and differentiate into terminally exhausted T cells (T_TEX_) (33, 46).

T_TEX_ cells typically co-express PD-1, LAG-3, TIM-3, CD160, and TIGIT (34, 38). However, they do not respond to immune checkpoint blockade and their proliferation potential is impaired (47).

T-cell exhaustion is a dynamic process from progenitor to terminally exhausted cells, characterized by different stages, each with distinct features. Understanding this process is necessary to designing more precise immunotherapy strategies. These approaches would help block the differentiation toward exhaustion and reverse certain stages of exhausted T cells (48).

### CD4^+^ T-Cell Subsets

**Figure 2:**
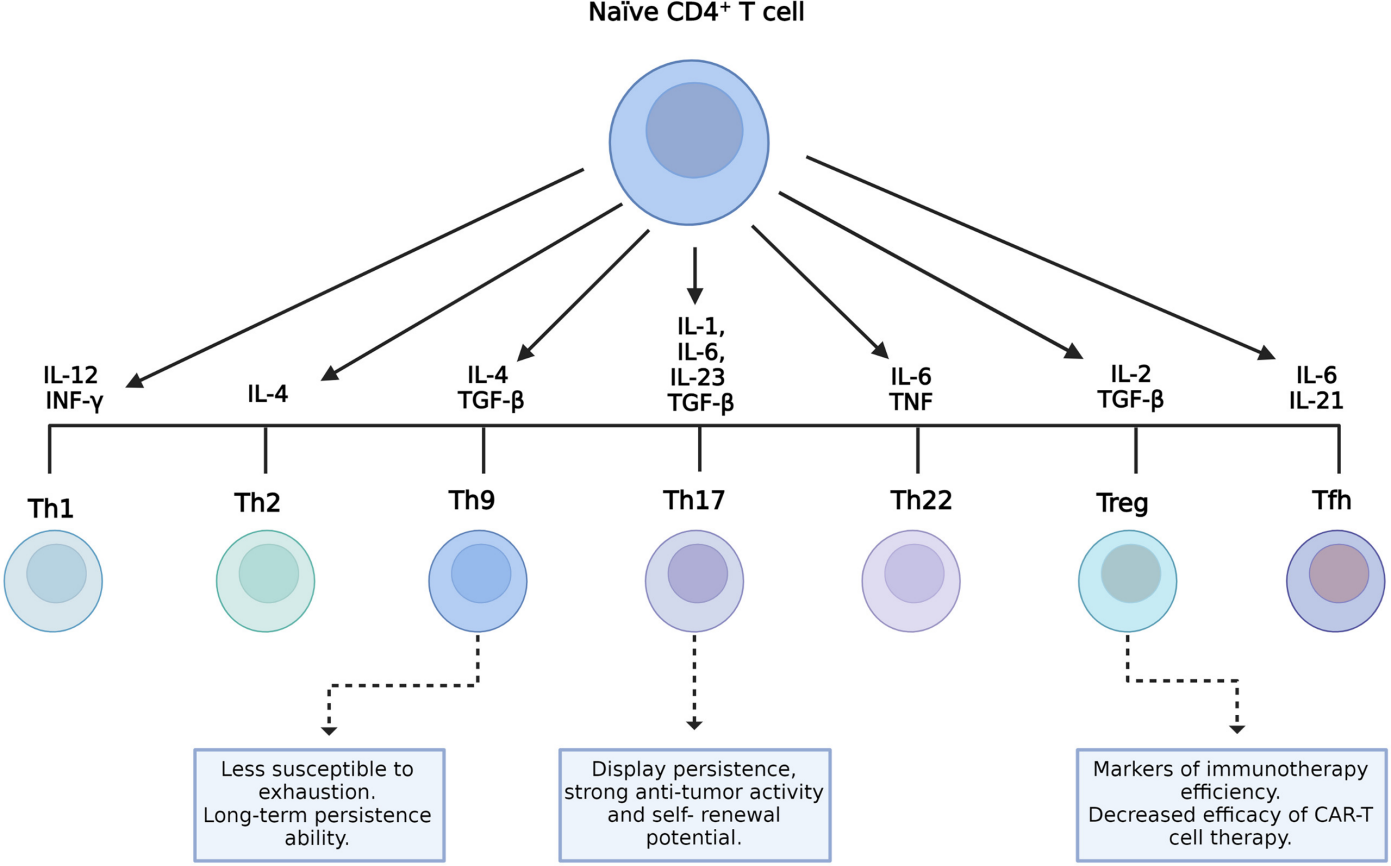
CD4^+^ T-cell subsets and their role on the final manufactured chimeric antigen receptor T-cell (CAR-T cell) product. Different environment cytokine combinations are required to generate different CD4^+^ T-cell subsets. Characteristics of T helper 9 (Th9) and Th17 cells with the potential to improve the efficacy of CAR-T cells and the role of regulatory T cells (Tregs) as indicators of therapeutic efficacy are shown.

CD4^+^ T cells can differentiate into several subpopulations, namely, T helper (Th) 1, Th2, Th9, Th17, Th22, follicular helper T cells (T_FH_), and regulatory T cells (T_reg_). Cell differentiation depends on the cytokines present in the environment and the strength of TCR signaling (**Figure 2**) (49). Each cell subset has particular characteristics and releases a cytokine cocktail that defines its functions. These roles can either be anti- or pro-inflammatory, related to protection, survival, or immune homeostasis (34). Several preclinical trial data have demonstrated that, in ACT with CAR-T cells, CD4^+^ T cells showed a direct antitumor activity comparable to cytotoxic CD8^+^ CAR-T cells (50–52). Moreover, the function of CD8^+^ CAR-T cells was characterized by exhaustion and apoptosis in the presence of antigen-specific TCR stimulation, whereas CD4^+^ CAR-T cells retained equivalent cytotoxicity despite TCR stimulation (50). CD4^+^ CAR-T cells helped augment the proliferation of CD8^+^ T cells, but they did not ameliorate the potency of CD8^+^ CAR-T cell effectors (51). Interestingly, in a murine model using CD28-based second-generation CAR-T cells, Th1 and Th2 cells released cytokines that led to different types of cytotoxicity (53).

In addition, tumor-specific Th9 cells are less susceptible to exhaustion, cause complete lysis of tumor cells, and persist longer given their unique hyperproliferative feature (**Figure 2**) (54). In patients undergoing ACT, resistance can rise due to the outgrowth of antigen-loss-variant (ALV) cancer cells (55). In a recent study, adoptively transferred tumor-specific Th9 cells have been shown to be able to eradicate established ALV-containing tumors (56). Human autologous mesothelin-specific CAR Th9 cells, but not regular or high doses of Th1+Tc1 CAR-T cells, were able to eradicate human ovarian cancer (OvCa) patient-derived xenograft (PDX) in humanized NSG (NOD scid gamma) mice (55). Interestingly, a recent study has shown that the intrinsic activation of CD4^+^ T cells potentiates the antitumor effects of Th9 cells upon adoptive transfer in mice and that human Th9 cell differentiation can be enhanced through STING activation (57). In addition, other studies found that STING agonists synergize with CAR-T cells, enhancing their ability to control tumor growth (58), and administration of the STING ligand cyclic guanosine monophosphate–adenosine monophosphate (cGAMP) improved the antitumor responses in models of melanoma and colon cancer (59, 60).

On the other hand, Th17 cells are characterized by their plasticity since they can transdifferentiate into other effector subsets, including Th1-like Th17 cells that express the transcription factor T-box-expressed-in-T-cells (T-bet), IL-17, and IFN-γ (61–63). The role of this cell subset in tumor immunity remains partly elucidated. According to some studies, Th17 cells can either promote or eliminate tumors depending on the context of the tumor (64, 65). Interestingly, several reports have shown that the antitumor activity of Th17 cells is related to their ability to recruit and activate cytotoxic T lymphocytes, natural killer (NK) cells, dendritic cells (DCs), and neutrophils into the tumor and also to the plasticity of Th17 cells to differentiate toward the Th1 phenotype that eliminates tumors *via* the secretion of IFN-γ (62, 66, 67). IL-17, one of the Th1-like Th17-related cytokines, is associated with a pro-tumorigenic role by controlling tumor angiogenesis, increasing cell proliferation, and preventing cell apoptosis (62). Although IL-17 shows some antitumor effects, it is IFN-γ rather than IL-17 from Th1-like Th17 cells that appears essential to an efficient antitumor response (68–70). Indeed, the combination of STING agonists with Th/Tc17 CAR-T cells increased the trafficking, persistence, and tumor control in a murine model of breast cancer (58). Furthermore, Guedan et al. reported the enhanced antitumor activity and increased persistence of CAR-T cells in a preclinical model of ACT using CAR Th17 cells engineered with an inducible T-cell co-stimulator (ICOS) domain (71). In recent years, it has become clear that Th17 cells display persistence, self-renewal potential, and the ability to drive potent antitumor responses (**Figure 2**) (65).

T_reg_ cells exert an immunosuppressive function and play a key role in maintaining immune homeostasis. They prevent unwanted immune reactions, such as autoimmunity and allergies. Usually, they express CD95^+^ and CD127^low^ (34). However, their presence in tumors is related to disease progression as they inhibit the antitumor immune response (**Figure 2**) (72).

The T_reg_/T_E_ cell ratio is a key marker of the efficacy of immunotherapy (71). Infiltrating CD4^+^ T_reg_ cells in solid tumors decrease the efficacy of CAR-T cell therapy (73). Deletion of the Lck-binding region within the CD28 endodomain, which is linked to IL-2 production, reverses T_reg_ cell-induced tumor infiltration and enhances the antitumor activity of CAR-T cells (73).

Given the diversity of CD4^+^ T-cell subpopulations and the cytokines they secrete, it is essential to characterize them in the final product of the CAR-T cell manufacturing process. This way, the conditions necessary to enrich less differentiated T cells in the final CAR-T cell product can be defined to improve their antitumor efficacy *in vivo* (74).

## Strategies to Improve the Persistence of CAR-T Cells

### CAR Architecture

**Figure 3:**
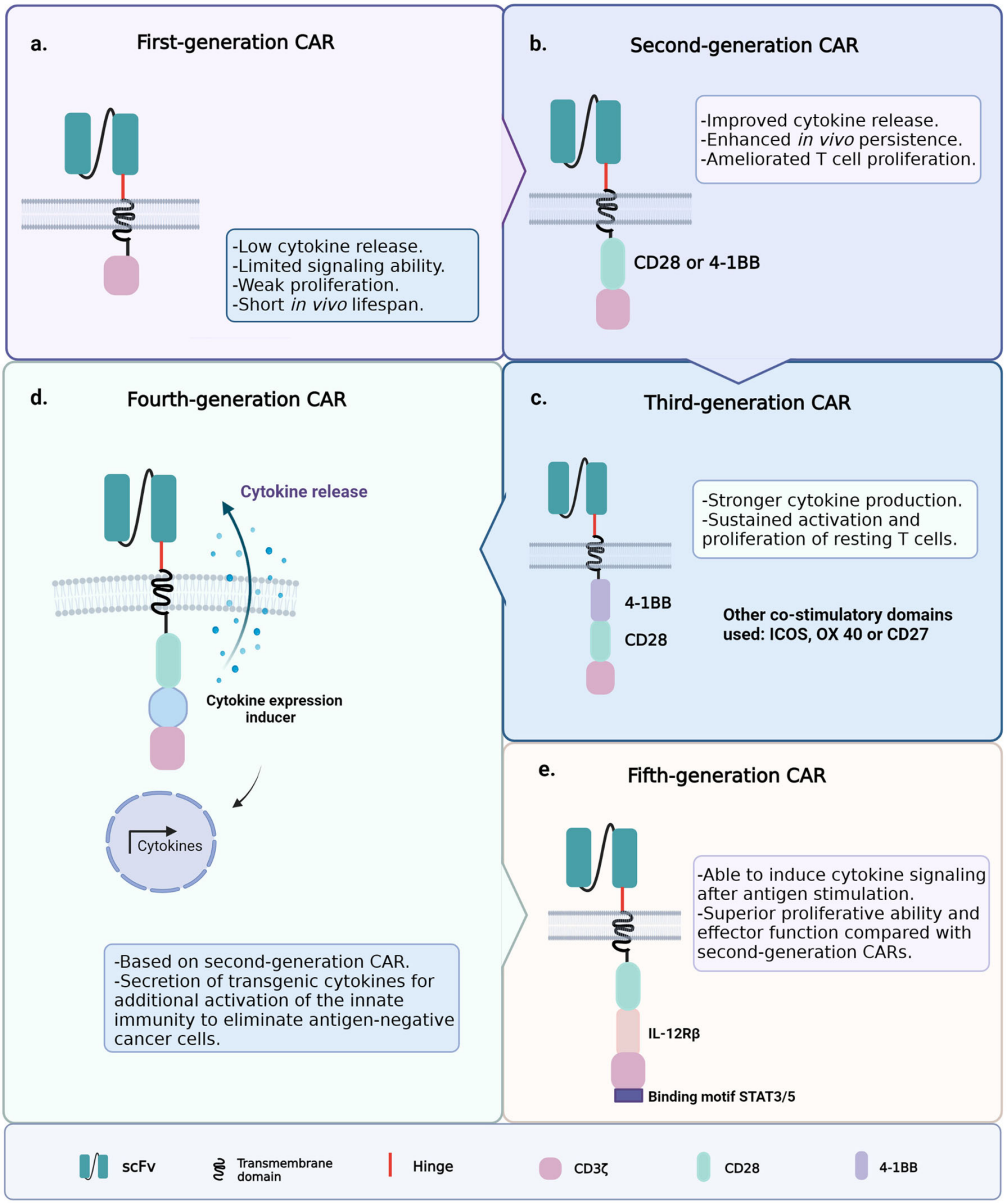
Generations of chimeric antigen receptor T-cell (CAR-T cell) construct designs. **(A)** First-generation chimeric antigen receptors (CARs) were composed of a single-chain variable fragment (scFv) specific for a cancer marker, hinge and transmembrane domains, and the cytoplasmic CD3ζ signaling domain. **(B)** Second-generation CARs included the coupling of a co-stimulatory signaling domain. **(C)** Third-generation CARs incorporated a second co-stimulatory signaling domain. **(D)** Fourth-generation CARs were based on the structure of the second-generation CARs, plus an inducible gene expression cassette encoding a transgenic cytokine. **(E)** Fifth-generation CARs contain an IL-2 receptor β-chain domain and a binding site for STAT3.

Since the introduction of ACT with CAR-T cells, the clinical outcomes have been hindered by the poor persistence of the engineered T cells; therefore, several CAR-T cell generations have been developed to improve cell persistence and functionality (75). The first generation of CAR-T cells had the simplest architecture, i.e., an extracellular single-chain variable fragment (scFv) specific for a cancer marker; hinge and transmembrane regions usually derived from the CH2–CH3 region of IgG1, IgG4, or CD8; and the cytoplasmic CD3ζ signaling domain (**Figure 3A**) (76, 77). Signaling through the CD3ζ domain did not suffice to prime resting T cells, and the first CAR-T cell generation could achieve neither sustained response nor cytokine release due to this limited signaling ability (78–80).

To improve T-cell signaling, the second generation of CAR-T cells included the coupling of a co-stimulatory signaling domain (e.g., CD28 or 4-1BB) that improves activation, enhances survival, and promotes the efficient expansion of the modified T cells (**Figure 3B**) (81, 82). Other common T-cell co-stimulatory molecules such as ICOS, CD27, and OX40 have been studied (83, 84). The currently approved FDA therapies Kymriah and Yescarta belong to this second-generation CAR-T cells. The experience gained from the application of second-generation CAR-T cells highlighted the relevance of the co-stimulatory molecule on the function and fate of the engineered cells within the TME (85). The addition of the 4-1BB (CD137) domain to CAR constructs promoted the induction of CD8^+^ T cells with increased respiratory capacity and heightened mitochondrial biogenesis, two characteristics of the least differentiated memory T cells (24, 25, 86). On the other hand, the inclusion of the CD28 co-stimulatory domain induced the expansion of T_EM_ lymphocytes with a gene signature of glycolytic metabolism (87, 88). Consistent with the above, chimeric antigen receptors (CARs) containing CD28ζ or 4-1BBζ are more likely to activate genes associated with the T_E_ or T_M_ phenotype, respectively (89). This is why CD28ζ-containing CAR-T cells persist about 30 days, while those with 4-1BBζ are found even 4 years after the ACT in some patients (88).

Third-generation CARs incorporated a second co-stimulatory signaling domain to achieve greater functional potency (**Figure 3C**) (90, 91). For example, the addition of the CD28 and OX40 domains to a CD3ζ chain leads to the sustained activation, proliferation, and effector function of resting T cells through the NFκB signaling pathway (85). Furthermore, ICOS-dependent signaling in CAR-T cells has been shown to result in an enhanced cell survival following the ACT. This evidence highlights the importance of testing novel CAR-T cell constructs to counter solid tumors and non-lymphoid hematologic malignancies; approaches to enhance CAR-T cell persistence remain an unmet medical need to date (71, 92). The design of fourth-generation CARs, known as T cells redirected for universal cytokine killing (TRUCKs), was based on the structure of the second-generation CARs. They contain an inducible gene expression cassette coding for a transgenic cytokine, such as IL-12 (IL-8, IL-9, IL-15, and IL-18 are still under investigation), to be delivered into the targeted tissue (**Figure 3D**) (91, 93). The accumulation of IL-12 can effectively recruit innate immune cells to the TME and attack antigen-negative cancer cells that CAR-T cells cannot recognize (94, 95). Recently, fifth-generation CARs have been studied and engineered based on the second-generation CARs. They contain an IL-2 receptor β-chain domain and include a binding site for STAT3 (**Figure 3E**) (96). This CAR construct induces a robust cytokine secretion through the activation of the JAK/STAT signaling pathway in the targeted tumor after antigen stimulation (91).

**Figure 4:**
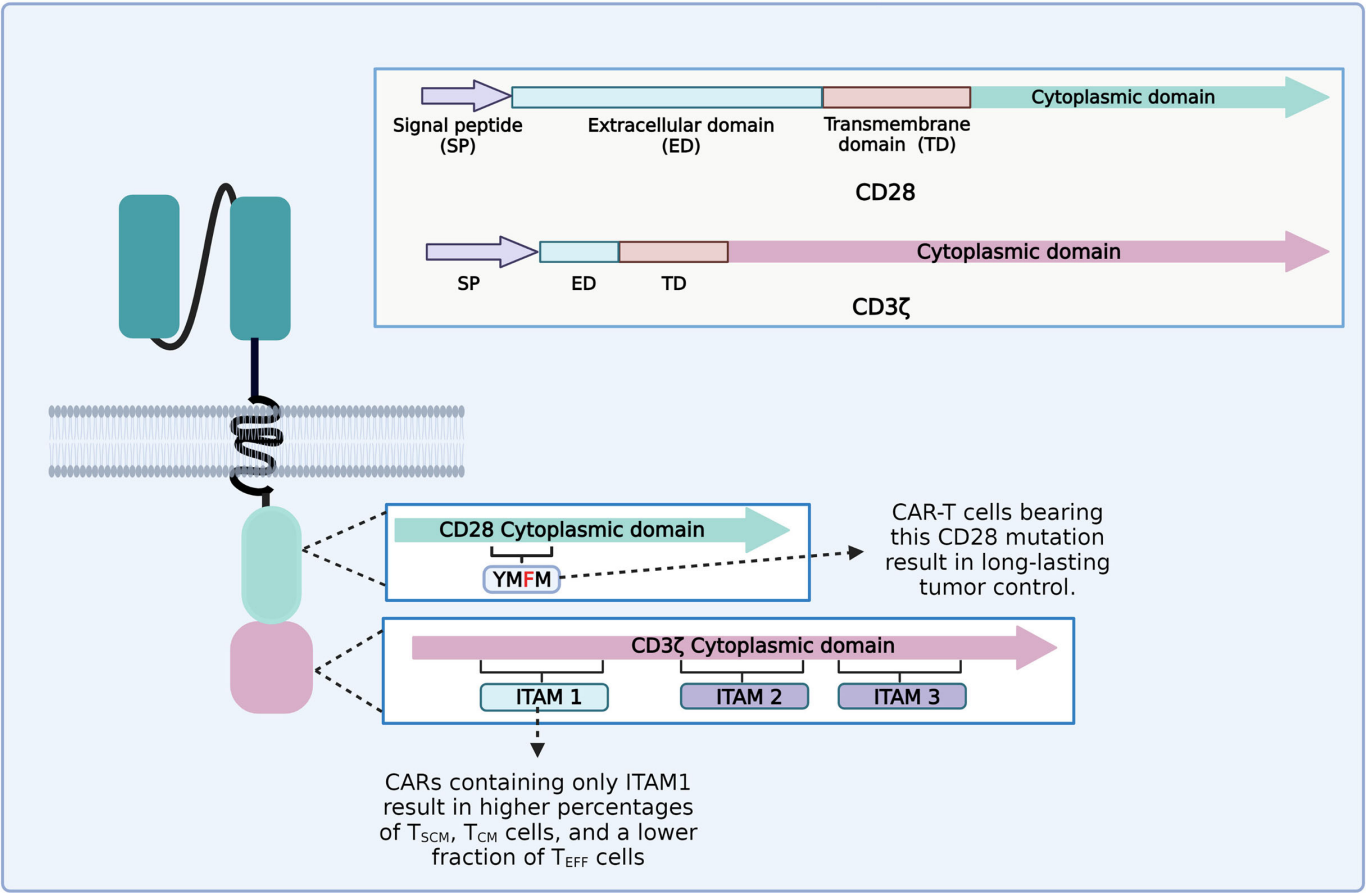
Diagram of the modifications on chimeric antigen receptor (CAR) cytoplasmic domains leading to enhanced tumor control and enrichment of less differentiated T-cell subsets related to better clinical outcomes.

It appears that each part of the CAR construct can influence the persistence and phenotype of CAR-T cells. For instance, the CD3ζ chain generally employed as a signaling domain on CAR constructs contains three immunoreceptor tyrosine-based activation motifs (ITAMs) (97). An ITAM domain consists of two consecutive YxxL/I motifs separated by a defined number of amino acids (YxxL/I-X6−8-YxxL/I) (98). TCR binding to the peptide–MHC complex leads to the activation of the Src family kinase Lck, which phosphorylates two tyrosine residues in each of the CD3ζ ITAMs (97, 99). CD3ζ ITAMs have different roles in the regulation of T-cell activation; for example, mutations of CD3ζ ITAM1 and ITAM2 significantly impaired signal transduction and induced cell death. However, mutation of CD3ζ ITAM3 did not induce cell death, but rather increased IL-2 secretion and MAPK phosphorylation (99, 100). CD28ζ-based CAR-T cells that only contain the ITAM1 domain resulted in higher percentages of T_SCM_ and T_CM_ and a lower fraction of T_EFF_ cells and yielded long-lasting and complete tumor remission in an *in vivo* animal model (**Figure 4**) (101). The CD28 cytoplasmic domain contains a YMNM motif that gets phosphorylated upon binding to CD80/CD86 ligands, which can bind to Grb2/Gads through the asparagine residue (102). Besides, the CD28 proline-rich regions can interact with Itk, Tec, Lck, Grb2/Vav, and filamin A (FLNA) (99, 103). CAR-T cells bearing a mutated CD28 domain (CD28-YMFM) promote a long-lasting antitumor control (**Figure 4**). Besides, CD28-YMFM CAR-T cells exhibit reduced differentiation and exhaustion and increased skewing toward the Th17 profile (104).

Several studies have shown that the extracellular spacer module (the hinge) significantly impacts the performance of CAR-T cell. Tumor membrane-proximal epitopes are best accessible to CARs with long spacers, while CARs with short spacers exhibit the highest activity against distal epitopes (105–107). These facts support the hypothesis that an optimal distance between T cells and target cells is required for CAR-T cells to be able to trigger an effective immune response. Therefore, a spacer is not required when the epitope is far from the T cells and, conversely, when the tumor epitope is very close to the membrane; the lack of a spacer region might result in a length inadequate for optimal T-cell activity (105). Recent advances have revealed that the TCR acts as a mechanoreceptor. The difference in the dimensions of the ligated peptide MHC (pMHC)/TCR complexes (~15 nm) and the surrounding molecules provides tensive forces that transiently bend the membrane around TCR microclusters. Conformational changes induced by the initial TCR signaling may thus act as a molecular spring to provide defined forces on the engaged TCRs, and such forces might be an amplifying source for T-cell activation (108). Thus, the intercellular space length between T cells and antigen-presenting cells (APCs) that leads to the best activation is equivalent to ~4 Ig-like domains (~15 nm) (108, 109). However, high-affinity TCR ligands can effectively induce TCR triggering even at large interspatial distances between T cells and APCs (109). There is evidence that the CD22-specific chimeric TCR signal strength and Ag sensitivity can be modulated by selecting target epitopes according to their distances from the cell membrane, allowing discrimination between targets with disparate Ag density (107).

Modified CARs have been developed to reduce the binding to soluble Fc gamma receptors (FcγRs); for instance, the CD19R(EQ) contains IgG4-Fc spacers carrying two mutations within the CH2 region (L235E and N297Q), and the CD19Rch2Δ incorporates a CH2 deletion. These CAR-T cells exhibit improved persistence and more potent CD19-specific anti-lymphoma efficacy in NSG mice (110). A novel class of CAR spacers with similar attributes to IgG spacers, but without unspecific off-target binding derived from the sialic acid-binding immunoglobulin-type lectins (Siglecs), was used to build a CAR directed against a membrane-proximal (TSPAN8) epitope of a pancreatic ductal adenocarcinoma model. *In vivo* settings using these CAR-T cells led to the generation of advantageous T_CM_ cells that released low levels of inflammatory cytokines and retained excellent tumor-killing functions (111).

Another important strategy consists in modulating the affinity to the scFv. A novel CD19 CAR (CAT), with a lower affinity than FMC63, showed higher *in vitro* proliferation and cytotoxicity and greater *in vivo* proliferative and antitumor activity compared with FMC63 CAR-T cells (112). In this sense, there is a minimal TCR affinity needed for T-cell activation and, additionally, a plateau to achieve maximal T-cell activity, revealing a TCR affinity threshold (113). Indeed, at low-density peptide ligands, the response is expected to be dominated by the low TCR-to-peptide interaction capable of using serial triggering to achieve the threshold required for T-cell activation. In contrast, high-affinity TCR-to-peptide interactions cannot achieve the activation threshold (114).

An additional strategy for optimizing the transferred construct includes the expression of non-coding RNAs. For instance, recent studies have shown that CAR-T cells engineered to express and deliver the RN7SL1 promote the expansion and effector memory differentiation of CAR-T cells characterized by high persistence and less exhaustion (115–118).

New concepts derived from the synthetic biology field for developing novel approaches in cell therapy are becoming appealing, such as the design of engineered cells harboring synthetic gene circuits able to biologically sense and compute signals derived from intracellular or extracellular biomarkers (119). These biological devices could ultimately be integrated into increasingly complex systems (119). The possibility of engineering T cells with synthetic systems responding to multiple inputs would benefit ACT with CAR-T cells and will probably open the door to the next generation of smarter self-decision-making CAR-T cells (120). For example, a generation of CAR-T cells that are only effective locally might also increase the choice of tumor targetable antigens. In this sense, incorporating the oxygen-sensitive domain (HIF-1a) could generate a CAR construct with gene expression induced by a low oxygen concentration, a characteristic of the TME (120).

### Enhance Expansion/Persistence by Vaccination

A multicenter phase I/II study of donor CD19-directed CAR-transduced Epstein–Barr virus (EBV)-specific cytotoxic T lymphocytes (CTLs) in pediatric patients with acute ALL showed that the use of donor EBV-specific CTLs to manufacture CD19CAR could enhance the CAR-T cell expansion/persistence after vaccination with EBV-specific peptides (121). A different approach to enhancing the CAR-T cell function against solid tumors is by directly boosting donor cells with a vaccine that interacts with the chimeric receptor *in vivo*. The idea is to attach a small target molecule (peptide or protein–ligand) of a CAR into the membrane of the APCs of lymph nodes using the amphiphile ligand (amph-ligand). In this way, the target epitope is displayed on the APC surface together with a native cytokine receptor. The amph-ligand strategy has safely expanded CAR-T cells *in vivo*, increased their functionality, and enhanced their antitumor activity in multiple models of solid tumors (122). A new approach uses a nanoparticulate RNA vaccine designed for wide delivery of the CAR antigen into lymphoid compartments throughout the body. This vaccine stimulates the presentation of the natively folded target of the CAR-T cells on resident dendritic cells to promote the cognate and selective expansion of CAR-T cells. This strategy improves the engraftment of CAR-T cells and the regression of large tumors in mouse models (123).

The upgrading of the previously described approaches requires more studies to evaluate the usefulness of combined CAR designs, including different generations of CARs and advanced vaccination strategies. In addition, to further improve these strategies, the CAR expression could be placed under the control of the TRAC locus, which has been shown to avert the tonic CAR signaling and delay the effector T-cell differentiation and exhaustion (122, 124).

### CAR-T Cell Therapy Combined With Oncolytic Viruses

Several preclinical studies have shown that oncolytic viruses (OVs) can synergize with CAR-T cells to overcome the multiple challenges that CAR-T cell therapy encounters in solid tumors by increasing CAR-T cell trafficking within the tumor and enhancing antitumor activity, as well as eliminating antigen-negative cancer cells (125, 126). In 2014, Nishio et al. armed an oncolytic adenovirus (Ad5Δ24) with the chemokines RANTES (regulated upon activation, normal T cells expressed and secreted) and IL-15 to enhance the trafficking and survival of a third generation of anti-GD2 CAR-T cells on a neuroblastoma xenograft model. They observed that intratumoral administration of this OV led to the improved persistence and migration of the infused CAR-T cells (127). Furthermore, the use of OV-IL15C, an oncolytic virus expressing the IL15/IL15Ra complex, enhanced the persistence of EGFR-CAR T cells that elicited strong antitumor responses in glioblastoma and improved this therapy in an immunocompetent mouse model (128). Another example of this strategy is T-SIGn, an oncolytic viral vector encoding IFNα, MIP1α, and CD80 that acts in synergy with anti-EGFR CAR-T cells. Tumor lysis induced by T-SIGn releases neoantigens and upregulates the antigen processing and presentation machinery to promote epitope spreading. Therefore, the virus reprograms the immunosuppressive TME into a pro-inflammatory one to attract and activate CAR-T cells and innate antigen-presenting cells, amplifying the antitumor response. This strategy was intravenously administered to eliminate pulmonary metastases in a murine model (129).

Despite the promising results obtained in murine models, this approach must be evaluated. Currently, only one ongoing clinical trial (NCT03740256) is being conducted in patients with human epidermal growth factor receptor 2 (HER2)-positive cancer. This study aimed to evaluate the safety and efficacy of anti-HER2 CAR-T cells combined with intratumoral injection of CAdVEC (an oncolytic adenovirus designed to enhance the antitumor immune response). This trial is on recruitment status and there are no results yet (130).

### Cell Culture Optimization

The culture medium used for the expansion phase of CAR-T cells impacts the cell performance *in vivo* (33). Components of the culture medium can influence not only the gene delivery but also the differentiation, proliferation, and potency of CAR-T cells (131, 132).

Usually, the culture medium is supplemented with serum of animal or human origin. Fetal bovine serum (FBS) is often used in research in a broad range of cell cultures as a source of nutrients and growth factors. However, it has several issues: firstly, FBS does not simulate the human microenvironment, which limits its translational application (131); secondly, it involves the risk of transmitting bovine spongiform encephalopathy and some viral infections; thirdly, it can promote the development of unwanted immunological reactions; and, finally, variations between the brands and batches of sera can affect the reproducibility of the experiments (131, 133, 134). Human serum (HS) does not contain any xenogeneic components and supplements the medium with trophogens and additional stimuli that favor cell growth and survival. However, the serum can inhibit cell growth (at high concentrations), is expensive, and there is a marked variability between different batches (131). Medvec et al. observed that expanding T cells in a medium without human serum improved their functionality and persistence (135).

Since many of the metabolites and growth factors required for cell proliferation originate in cells such as erythrocytes, platelets, and endothelial cells, some researchers have studied whether extracts derived from these cells, obtained from whole blood fractions, can support the differentiation and proliferation of T cells (131, 133). For example, blood platelets contain strong mitogens such as growth factors, chemokines, and cytokines (136). Recent studies have provided support that the human platelet lysate (HPL) allows the expansion of CAR-T cells and increases the percentage of T_CM_ cells in the final product compared to those obtained in media supplemented with FBS or HS (136). This observation suggests that HPL-supplemented media for culturing CAR-T cell improves the cell functionality *in vivo* and enriches the T_CM_ cell subset associated with increased cell persistence in patients following ACT (136). The impact of HPL-exposed CAR-T cells was evaluated *in vivo* in a mouse xenograft model. The cell proliferation and antitumor effects were more significant compared to those of CAR-T cells cultured in media supplemented with FBS or HS (137).

Ghassemi et al. found that, among other alternatives, the serum used for CAR-T cell expansion culture can be substituted by Physiologix™ (Phx), an extract of growth factors obtained from whole blood. Compared to CAR-T cells expanded with HS-supplemented media, CAR-T cells cultured in Phx-containing media displayed increased transduction efficiency, as evidenced by their *in vitro* cytotoxic activity and superior *in vivo* cell survival ability in neuroblastoma models. Additional metabolomic analyses of the composition of Phx showed a modest enrichment in carnosine, a dipeptide composed of the isomers β-alanine and l-histidine. Carnosine is a critical factor that improves the CAR transduction efficiency in activated T cells; it can also decrease the media acidification and induce a glycolytic-to-oxidative metabolic change, a characteristic related to better antitumor effects (131). Smith et al. demonstrated that xeno-free CTS™ Immune Cell Serum Replacement allows the efficient expansion of gene-modified T cells with similar yields to those generated when FBS or HS was used as a supplement (138). Moreover, as an alternative to the expensive serum-free specific culture media, acellular Wharton’s jelly can be utilized as a supportive substance; furthermore, it increases the memory properties of T cells (139).

Other approaches that have been studied include reducing the length of the cell expansion phase and the effect of RetroNectin on T cell culture. The protocols for T-cell engineering routinely expand T cells *ex vivo* for 9–14 days. However, Ghassemi et al., in 2018, reported that CAR-T cells targeting CD19 (CART19) expanded for 3–5 days proliferated more and showed greater cytotoxic ability *in vitro*, as well as in a murine xenograft model of ALL, showing that the antileukemic activity inversely correlated with the *ex vivo* culture time. In addition, these cells persisted longer and showed more robust antitumor activity in a murine model (140). A recently published work has shown that quickly generated (24-h expansion) non-activated CAR-T cells exhibited higher *in vivo* antileukemic activity per cell than the activated CAR-T cells produced using the standard protocol. The former protocol for the rapid manufacturing of CAR-T cells may reduce the production costs and broaden their applicability. However, immunosuppressive factors in the TME may hinder the ability to generate functional CAR-T cells using this approach (141). The facts previously described must be considered relevant in the microenvironment of solid tumors.

On the other hand, RetroNectin (a recombinant human fibronectin fragment containing the VLA-4 and VLA-5 binding domains) is generally used to enhance the transduction efficiency given its ability to co-localize viral vectors and cells of interest, such as hematopoietic progenitor cells and T lymphocytes (142, 143). When RetroNectin was used in conjunction with anti-CD3 monoclonal antibodies (mAbs), it also enhanced T-cell expansion while preserving the CD45RA^high^ CCR7^high^ phenotype, characteristic of T_N_ and T_CM_ cells (144–147). RetroNectin influences the CD4^+^/CD8^+^ composition of T-cell products by inhibiting the apoptosis of CD8^+^ T cells and shifting the cell composition toward a cytolytic phenotype over *in vitro* culture (144) and during their *in vivo* persistence (148).

### Cytokines Used to Yield Undifferentiated CAR-T Cells

Modulation of the interleukin cocktails can affect the memory functions of T cells and are used as an alternative approach to increase the efficacy of CAR-T cell therapy (144, 149–152). Cytokines are biologically active peptides that act by binding to their specific receptors located on cell surfaces (153). Interleukins are a subgroup of cytokines that allow communication between cells of the immune system; they determine processes such as the activation, differentiation, maintenance, function, and proliferation of immune cells (154). The interleukins chosen for the manufacturing process influences the T-cell proliferation and differentiation in CAR-T cell cultures (144). The most used and studied interleukin is IL-2, which has an essential role in the manufacturing process of CAR-T cells since it stimulates cell proliferation and maintains cell viability during the expansion phase (153). However, the stimulation of T cells with IL-2 favors the differentiation of short-life-span and exhausted cells because IL-2 induces a switch to glycolysis, a feature of T_E_ cells (33, 155).

The substitution of IL-2 with other γ-chain cytokines such as IL-7, IL-15, and IL-21 plays a crucial role in the functionality, homeostasis, differentiation, and expansion of T cells and allows obtaining more significant proportions of less differentiated lymphocytes (156). There is evidence that the culture of CAR-T cells in the presence of IL-15 reduces the activity of mTORC1 and conserves the stem cell memory phenotype that has higher antitumor activity and proliferative ability (33, 155). Some studies have shown that, during the expansion phase of CD28-based CD19 CAR-T cells, a mixture of IL-7 + IL-15 increased the number and proportion of a T-cell subpopulation with T_SCM_- and T_CM_-like phenotypes. Moreover, these CAR-T cells showed higher expansion and effector function abilities and more significant migration to secondary lymphoid organs, leading to longer cell persistence and antitumor activity *in vivo* (149, 157, 158).

Moreover, IL-21 is another member of the γ-chain cytokine family that has shown favorable effects on the T-cell expansion process. Loschinski et al. found that exposure of T cells to IL-21 reduced the glycolytic activity and increased the fatty acid oxidation (FAO), a pathway essential for T_CM_ generation (156). Furthermore, some studies have reported that a mixture of IL-21 + IL-4 + IL-7 added to the culture media maintained the memory phenotype and reduced the expressions of inhibitory receptors including PD-1 and TIM3 in CAR-T cells (159). The combination of IL-12 + IL-7 or IL-21 in the *ex vivo* cell expansion process yielded CD8^+^ T cells with enhanced persistence in a NOD/SCID/γc^−/−^ mouse model (150). IL-12 is a non-γ-chain cytokine important in regulating T-cell differentiation and memory generation (160).

### Metabolic Reprogramming of CAR-T Cells

The metabolic requirements of T cells depend on their degree of activation, differentiation, and functionality. For example, cells in a quiescent state, such as T_N_ cells, rely on a catabolic metabolism of low-energy consumption, which uses the oxidation of fatty acids, amino acids, and glucose as energy sources, mainly through oxidative phosphorylation pathways (161). However, upon cell activation, the metabolism becomes highly glycolytic to generate the intermediate biomolecules required for cell proliferation. Specific metabolic and epigenetic changes must occur in cells in order to proliferate and differentiate. As can be seen, metabolism is intimately linked to cell activation, proliferation, migration, and function, and therefore to the very fate of T cells (85, 161, 162).

As for the metabolic conditioning of CAR-T cells, the CAR design itself can define their metabolism and functionality. In this sense, it is known that the CD28 co-stimulating domain increases the glycolytic metabolism and differentiation of T cells toward the T_EM_ cell subpopulations. In contrast, the 4-1BB co-stimulating domain increases oxidative metabolism and mitochondrial biogenesis, promoting differentiation to the T_CM_ phenotype, characterized by improved cell proliferation and persistence (88, 161). Hence, it can be concluded that promoting a low metabolic activity over the CAR-T cell manufacturing process could favor the production of T cells with a less differentiated phenotype, which would have greater longevity and antitumor potential. Conversely, a high metabolic activity could favor an effector lymphocyte enrichment; therefore, following infusion into patients, these cells would be quickly depleted, resulting in reduced antitumor activity (162).

### Strategies to Target the T-Cell Metabolism

While studying the metabolome and proteome of activated CD4^+^ T_N_ cells, Geiger et al., in 2016, discovered that increasing the levels of l-arginine in the culture medium induced a metabolic shift from glycolysis to oxidative phosphorylation. This change promoted T-cell differentiation toward a T_CM_-like phenotype characterized by the expressions of CCR7 and CD62L (163).

Another approach is inhibiting the glycolytic metabolism using drugs or expanding cells in a medium with low glucose concentrations; for example, in 2013, Sukumar et al. used 2-deoxy-d-glucose, a hexokinase 2 inhibitor, to limit the glycolytic metabolism of CD8^+^ T lymphocytes. This strategy increased the development of T_M_ cells. Additionally, the researchers found that inhibition of the glycolytic pathway was associated with the expression of transcription factors that drive cell differentiation into the memory phenotype (164). The PI3K/AKT/mTor pathway is essential to regulate T-cell differentiation and memory generation. Its activation promotes the expression of the *GLUT1* gene, a glucose transporter, which promotes glycolytic metabolism by increasing glucose intake from the medium. Consequently, it facilitates the differentiation of T cells into an effector subset (162). The effect of this pathway inhibition on CAR-T cells has been studied. In 2018, Perkins et al. expanded BCMA-directed CAR-T cells together with a PI3K inhibitor to investigate its activity *in vivo*. The adoptive transfer of these CAR-T cells resulted in complete long-term tumor regression in animal models of Burkitt lymphoma and multiple myeloma. The animals were even immune to a re-challenge with tumor cells. In addition, the phenotypic analysis of these BCMA-directed CAR-T cells showed a high frequency of CD8^+^CD62L^+^ T cells. These results suggest that PI3K inhibition during *ex vivo* cell expansion generates a product with better antitumor efficacy *in vivo* (165). Similarly, in 2019, Zhang et al. demonstrated that the culture of epithelial cell adhesion molecule (EpCAM)-directed CAR-T cells with the AKT inhibitor MK2206 did not affect cell proliferation or viability. However, the AKT inhibitor prevented the terminal differentiation of CAR-T cells. These cells exhibited higher expansion and antitumor efficacy in an animal model of colon cancer. Also noteworthy was the finding that AKT inhibition increased the CAR rate expression (166).

Moreover, there is evidence that crosstalk between the Wnt pathway and IL-12 signaling inhibits the T-bet and mTOR pathways and impairs memory programming, which can be recovered in part by rapamycin (167). Furthermore, mTOR inhibition in activated T_N_ cells using a high concentration of rapamycin or TWS119 (an activator of the Wnt-β/catenin pathway) induced the generation of a T_SCM_ phenotype. The inhibition of the pathway induced a switch in the metabolism of T cells characterized by an increase in FAO. The T_SCM_ subpopulation exhibited superior functional characteristics and a more remarkable repopulation ability after adoptive transfer (168).

In 2016, the study of Bengsch et al. revealed that the peroxisome proliferator-activated receptor-gamma co-activator 1-α (PGC-1α) is a central regulator of oxidative phosphorylation. Moreover, when PGC-1α was overexpressed in T_EX_ cells, it corrected the dysregulated mitochondrial function, improving metabolic fitness and effector function (169). More recently, Dumauthioz et al. enhanced the mitochondrial biogenesis in CD8^+^ T cells by overexpressing PGC-1α and observed that this strategy improved the antitumor effect by promoting the generation of CD8^+^ memory T cells (170).

Furthermore, some reports have demonstrated that PD-1 blocking in CAR-T cells improved tumor control and overall cell survival. Moreover, the PD-1 inhibition in T cells led to a metabolic switch from glycolysis toward an increased FAO; these cells exhibited enhanced survival and similarities to memory T cells (171).

## Conclusion

**Figure 5:**
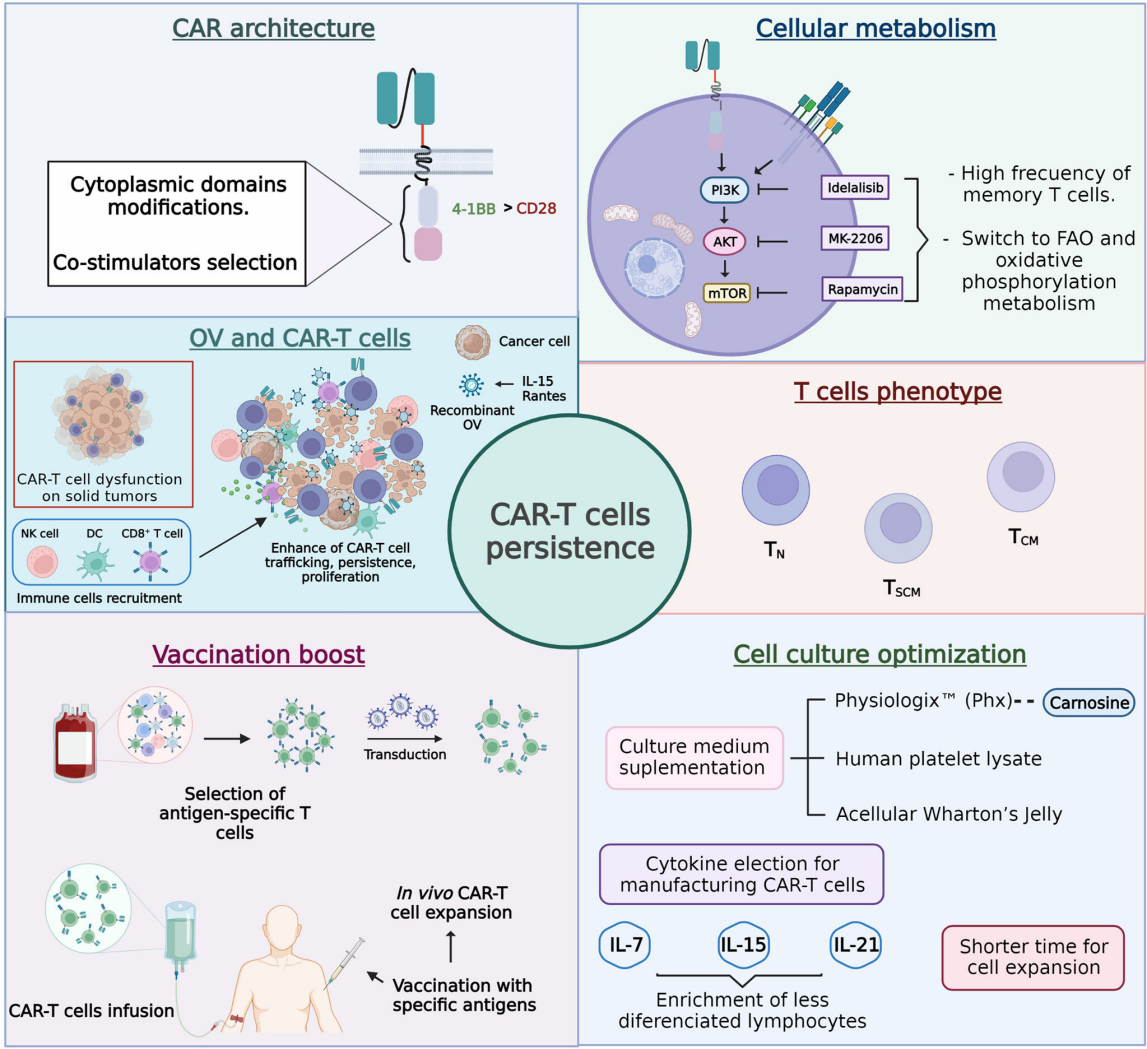
Approaches designed to improve chimeric antigen receptor T cell (CAR-T cell) persistence.

Cancer-specific chimeric antigen receptor (CAR) T cells have emerged as one of the most promising immunotherapies to target various types of cancer. However, many barriers must still be overcome to generate highly successful clinical outcomes. One of these unmet needs relates to the persistence of CAR-T cells, and multiple strategies focused on CAR’s architecture, cellular metabolism, T-cell phenotype, vaccination boost, and cell culture optimization are under development to improve it (**Figure 5**).

In agreement with the described facts, a plausible strategy to extend the *in vivo* persistence of CAR-T cells could require combinatory approaches, mainly when the therapy is directed against solid tumors because their hostile microenvironment induces the dysfunction of T cells, making the cell persistence requirements much higher. In this sense, understanding the underlying knowledge supporting each approach under study is extremely relevant. This way, rational combinatory strategies pursuing synergistic antitumor effects *in vivo* could be better chosen. Finally, it is essential to keep in mind that most of the reviewed approaches come from NSG mice research. Although this is the acknowledged model to assess cellular therapeutic efficacy, some factors cannot be accurately predicted, such as the interactions of CAR-T cells with the TME that could directly affect the persistence of CAR-T cells.

In this review, we summarized some of the approaches developed to circumvent the CAR-T cell short persistence barrier and offered ideas to tackle this hurdle to those researchers who have begun to work on CAR-T cell production.

## Author Contributions

GLC and CRS wrote and edited the manuscript. All authors reviewed, made an intellectual contribution, and approved the submitted version.

## Funding

GLC received financial support from the project entitled “Investigación Orientada a la Implementación de Buenas Prácticas para la Aplicación Clínica de Terapias Celulares. Modelo: tph en Bogotá” (Research Oriented to the Implementation of Good Practices for the Clinical Application of Cellular Therapies. Model Tph in Bogotá) (code BPIN2016000100035; agreement 0182 of 2018), signed with the District Health Financial Fund. CU was financed by resources of the “Colombia Científica” program of the Ministry of Science and Technology allocated to the Pontificia Universidad Javeriana. “Ecosistema Científico” call (contract no. FP44842-221-2018) and DreemBio. BAC was funded by the IDCBIS institute. CRS was financed with resources transferred from the District Health Financial Fund (FFDS, by its abbreviation in Spanish) to IDCBIS, according to Resolution 515 of April 12, 2021, issued by the District Health Secretariat.

## Conflict of Interest

The authors declare that the research was conducted in the absence of any commercial or financial relationships that could be construed as a potential conflict of interest.

## Publisher’s Note

All claims expressed in this article are solely those of the authors and do not necessarily represent those of their affiliated organizations, or those of the publisher, the editors and the reviewers. Any product that may be evaluated in this article, or claim that may be made by its manufacturer, is not guaranteed or endorsed by the publisher.
