# Supplementary material for: CAR-T Cell Performance: How to Improve Their Persistence?
Source: Front Immunol. 2022 Apr 28;13:878209. doi: 10.3389/fimmu.2022.878209 (PMC9097681; doi:10.3389/fimmu.2022.878209)
Supplement: Supplementary file 1 [file Table_1.docx]

| T-cell subset | Surface marker expression | References |
| --- | --- | --- |
| Naïve T-cells | CD3^+^, CD45RO^-^, CCR7^+^, CD45RA^+^, CD62L^+^, CD27^+^, CD28^+^, IL7Rα^+^(CD127), CD95^-^, CD31^+/-^, CD122^-^, KLRG1^-^, CXCR4^+^. | ^23,25,29,34,36,57^ |
| Stem cell memory  T-cells | CD3^+^, CD45RO^-^, CCR7^+^, CD45RA^+^, CD62L^+^, CD27^+^, CD28^+^, IL7Rα^+^(CD127), CD95^+^, Bcl2^+^, CD122^+^, KLRG1^-^, CXCR3^+^, CD11a^+^, CD31^-^, LFA-1^+^, LFA-3^+^, CXCR4^+^. | ^23,25,29,34,57^ |
| Central memory  T-cells | CD3^+^, CD45RO^+^, CD45RA^-^, CCR7^+^, CD27^+^, CD28^+^, CD62L^+^, IL7Rα^+^(CD127), CD95^+^, CXCR3^+^, LFA-1^+^, LFA-3^+^, CXCR4^+^, CD31^-^, CD122^+^, IL-18Rα^+^, KLRG1^+/-^, CXCR5 ^-/+^. | ^23,25,29,34,57,58^ |
| Effector memory  T-cells | CD3^+^, CD45RO^+^, CD45RA^-^, CCR7^-^, CD62L^-^, IL7Rα^-/+^ (CD127), CD27^-/+^, CD28^-/+^, CD122^+^, CD95^+^, KLRG1^+^, LFA-1^+^, LFA^-^3^+^, CD161^-/+^, IL-18Rα^+^, CD57^+^, CXCR3^+^, CXCR4^+^, CCR5^-/+^, IL7Rα^-/+^(CD127), CD31^-^. | ^23,25,29,34,57,58^ |
| Tissue resident memory  T-cells | CD62L^-^, CD69^+^, CD103^+^, CD101^+^, CD25^-^, CD38^-^, HLA-DR^-^.  Skin: CLA^+^, CCR4^+^, CCR6^+^, and about 50% express CCR5^+^ and CXCR3^+^.  Gut: CD69^+^, CCR6^+^, CCR9^+^, CD49d^+^.  Lung: CD49a^+^, PSGL^-^1^+^, CCR5^+^, CXCR3^+^, CCR6^+^ | ^33,34,59^ |
| Effector  T-cells | CD95^+^, CD122^+^, CD45RA^+^, CD45RO^-^, CCR7^-^, CD62L^-^, IL7Rα^-^ (CD127), CD27^-^, CD28^-^,  LFA-1^+^, LFA^-^3^+^, CX3CR1^+,^ CCR5^+^. | ^29,34,42^ |

**Table S1.** Surface markers associated to T-cell differentiation stages
